# Preventive but Not Therapeutic Topical Application of Local Anesthetics Can Inhibit Experimental Epidermolysis Bullosa Acquisita in Mice

**DOI:** 10.3389/fimmu.2021.750160

**Published:** 2021-10-12

**Authors:** Lifang Wen, Xiaoru Dong, Qing Li, Gabriele Schramm, Bing Zhang, Detlef Zillikens, Ralf J. Ludwig, Frank Petersen, Xinhua Yu

**Affiliations:** ^1^Department of Basic Medical Science, The Medical College of Xiamen University, Xiamen, China; ^2^Priority Area Asthma & Allergy, Research Center Borstel, Airway Research Center North (ARCN), Member of the German Center for Lung Research Deutsches Zentrum für Lungenforschung (DZL), Borstel, Germany; ^3^Clinical Laboratory, Boai Hospital of Zhongshan, Zhongshan, China; ^4^Department of Dermatology, University of Lübeck, Lübeck, Germany; ^5^Lübeck Institute of Experimental Dermatology and Center for Research on Inflammation of the Skin, University of Lübeck, Lübeck, Germany

**Keywords:** epidermolysis bullosa acquisita, autoantibodies, scratching, dyclonine hydrochloride, itching, anesthetics

## Abstract

Epidermolysis bullosa acquisita (EBA) is an autoimmune blistering disorder characterized and caused by autoantibodies against type VII collagen (COL7). Although it has been noticed that EBA in both patients and mice is associated with an increased scratching, it is not clear whether and how the scratching contributes to disease manifestation. Hence, we here aimed to validate this clinical observation and also to investigate the potential contribution of increased scratching in EBA pathogenesis in mice. Longitudinal assessment of scratching behavior revealed an increased frequency of scratching as early as 12 hours after injection of anti-COL7 IgG into the skin of mice. Subsequently, scratching events became even more frequent in mice. In contrast, mice injected with a control antibody showed an unaltered scratching behavior throughout the observation period. Based on these observations, we hypothesized that mechanical irritation may promote the induction of inflammation in experimental EBA. To challenge this assumption, the local anesthetic dyclonine hydrochloride was topically applied before injection of anti-COL7 IgG. Dyclonine hydrochloride reduced the scratching events and impaired clinical disease manifestation. In therapeutic experimental settings, i.e. administration of the local anesthetic 24 hours after injection of anti-COL7 IgG, dyclonine hydrochloride only inhibited the scratching behavior, but had no significant effect on clinical disease development. In addition, eosinophils were detected in the skin before the injection of anti-COL7 IgG and significantly increased 48 hours after the antibody injection. Collectively, our results suggest that scratching behavior contributes to the initiation phase of disease manifestation in experimental EBA.

## Introduction

Autoimmune bullous dermatoses (AIBD) represent a group of acquired organ-specific autoimmune diseases mediated by autoantibodies targeting proteins which are essential for integrity of skin and mucous membranes (1, 2). Within this group, epidermolysis bullosa acquisita (EBA) is a subepidermal blistering disorder characterized and caused by autoantibodies against type VII collagen (COL7) (3, 4). To better understand the pathogenesis of EBA, several mouse models have been established either by immunizing mice with recombinant murine COL7 (mCOL7) (5) or by transfer of antibodies against COL7 into mice (6–8). With these valuable experimental models, considerable progress has been achieved in our understanding of how autoantibodies against COL7 cause inflammation and tissue damage (9). So far, several cellular and molecular components have been demonstrated to be indispensable in the pathogenesis of experimental EBA. In general, EBA has been shown to be associated with MHC alleles, in both patients and experimental models (10, 11). Development of autoantibodies targeting COL7 depends on a CD4-dependent B cell response (12), and half-lives of these autoantibodies are controlled by the neonatal Fc receptor (13). After binding to its target antigen, autoantibody-induced inflammation and blistering is modulated by several cell types, such as neutrophils (14, 15) and T cells (16), as well as several molecular pathways, including complement activation (6, 17), reactive oxygen species (18), specific proteases (19), lipid mediators (20), signaling molecules (21), and several cytokines (22, 23).

It has been noticed that some AIBD such as bullous pemphigoid (BP) and EBA are associated with an increased scratching and skin lesions often occur in areas which are accessible to scratching (2, 24). In experimental EBA models, we also noted that i) there is an increased frequency of scratching in diseased mice and ii) skin lesions usually do not develop in skin areas which mice cannot directly access. However, a simple association of a certain factor or event with experimental EBA does not necessarily mean that this also contributes to the pathogenesis of the disease. Activation of mast cells, which typically occurs during the early phase of experimental EBA, does not contribute to disease development. Moreover, upregulation of the two proinflammatory molecules S100A8 and S100A9, which are seen in experimental EBA and in many other neutrophil-dominated inflammations, is without consequence for the manifestation of disease symptoms (25, 26). In this study, we hypothesized that scratching behavior contributes to disease manifestation of experimental EBA. To verify this hypothesis, we herein first aimed to validate the clinical observation of an increased scratching frequency in mice with experimental EBA. By contrasting the frequency of scratching events between mice injected with anti-COL7 IgG to mice injected with control antibody, we observed increased scratching events in mice with EBA. Hence, we next evaluated if increased scratching contributes to clinical disease manifestation in experimental EBA. To reach this aim, we applied dyclonine, a topical anesthesia acting through sodium channel inhibition (27, 28), onto mouse skin at different phases of the development of experimental EBA. Our results suggest that the scratching behavior contributes to the initiation phase, but not the late phase, of the development of the skin disease.

## Materials and Methods

### Mice

Female Balb/c mice were purchased from Shanghai SLAC Laboratory Animal Co., Ltd. (Shanghai, China). All mice were housed in the animal facility of Xiamen University with a *12*-*hour*light-dark cycle. Mice were held at specific pathogen-free conditions and fed standard mouse chow and acidified drinking water ad libitum. All experiments were approved by the Institutional Animal Care and Use Committee of Xiamen University (XMULAC20170196).

### Antibodies

Rabbit anti-mCOL7c antibodies and rabbit control IgG were prepared as described (6). Briefly, New Zealand White rabbits were immunized subcutaneously with recombinant mCOL7c protein (amino acid residue at position 757-967) emulsified in Freund’s complete adjuvant. The animals were boosted twice with mCOL7c protein emulsified in incomplete Freund’s adjuvant. Sera from the immunized rabbit were obtained and used for the isolation of total IgG by Protein G Sepharose Fast Flow affinity column chromatography. Control rabbit IgG was isolated from sera of non-immunized rabbits. The binding specificity of rabbit anti-mCOL7 IgG to murine skin was shown in **Supplementary Figure 1**.

### Induction and Evaluation of Experimental EBA

Induction of experimental EBA was performed as described previously (26, 29) with minor modifications. Briefly, 0.5 mg rabbit anti-mCOL7 IgG or control rabbit IgG in 50 µl PBS was injected i.d. into the base of the ear. Mice were scored blindly for clinical symptoms at 24 h and 48 h after injection of antibodies. Disease severity of ear skin was determined using a previously described method (29). Briefly, severity of disease was expressed as percentage of the surface area of the ear affected by skin lesions including blisters, erosions, crusts and alopecia. At the end of the experiment, ear skin samples were collected for histological evaluation.

### Histology

Mouse ears were fixed in 4% buffered formaldehyde and embedded in paraffin. Five µm-thick paraffin sections were prepared and used for histological evaluation using hematoxylin and eosin (H&E) staining. Eosinophils were identified on the H&E-stained paraffin-embedded skin sections. For the identification of basophils, sections of ear skin of anti-COL7 IgG-treated or control IgG-treated mice, respectively, were stained with the basophil-specific rat anti-mMCP-8 antibody (clone TUG8, Biolegend) as described elsewhere (30). Sections of liver tissue from *Schistosoma mansoni*-infected mice were used as positive staining control. Briefly, deparaffinised tissue sections were treated consecutively with Tris-EDTA-buffer, pH 9.0, at high temperature for antigen retrieval, with 0.9% H_2_O_2_ in methanol for blocking of endogenous peroxidase activity, and with normal rabbit serum for blocking unspecific antibody binding. For basophil staining, all antibodies (anti-mMCP8, isotype control rat anti-mouse IgG2a (BD) and the secondary biotinylated rabbit anti-rat antibody (Vector Laboratories)) were diluted 1:100 in PBS. Detection of antibody binding was performed using the Vectastain ABC Peroxidase Kit and the substrate DAB (Vector Laboratories) according to the manufacturer’s instructions. Mayer’s hematoxylin (Merck) was used diluted 1:2 for counter staining. Pictures were taken using an Olympus BX51 microscope. Quantification of eosinophils was performed by counting them in whole section.

### Application of Dyclonine Hydrochloride

Dyclonine hydrochloride (dyclonine, Sigma-Aldrich, Darmstadt, Germany) was dissolved in 71.4% glycerin at a concentration of 1%. For treatment, 50 μl 1% dyclonine, a concerntration which has been shown effective in patients (27, 28), was topically applied onto each mouse ear every five hours, while 71.4% glycerin was used as solvent control. Topical application of dyclonine started five min before (prevention) or 24 h after (therapy) the antibody injection and was maintained until the end of the experiment. In total, 10 and 5 applications of dyclonine were performed for preventive and therapeutic treatment, respectively.

### Quantification of Scratching Behavior

In this acute antibody transfer-induced experimental EBA, skin lesions are visible approximately 20 hours after the injection of pathogenic antibodies, and disease severity increases over time and reachs the peak 48 hour after the injection of antibody (29). Therefore, scratching events were determined in a blinded fashion at three time points: 12, 30 and 48 h after the antibody injection, which represent three different phases of the development of disease. For each time point, the number of scratching events over 60 min observation period was counted for and the scratching frequency was presented as the number of scratching events per hour.

### Statistical Analysis

The Kolmogorov-Smirnov normality test was performed to analyze if quantitative variables were normally distributed. All statistical analysis were performed with GraphPad Prism statistical software (GraphPad Software Inc., version 5.01, La Jolla, CA, USA). To calculate the *P* values, quantitative data in normal distribution were compared using the student t-test, otherwise the Mann-Whitney U-test was used. *P*<0.05 was considered as statistically significant.

## Results

### Injection of Anti-mCOL7 IgG Promoted Scratching Behavior

**Figure 1:**
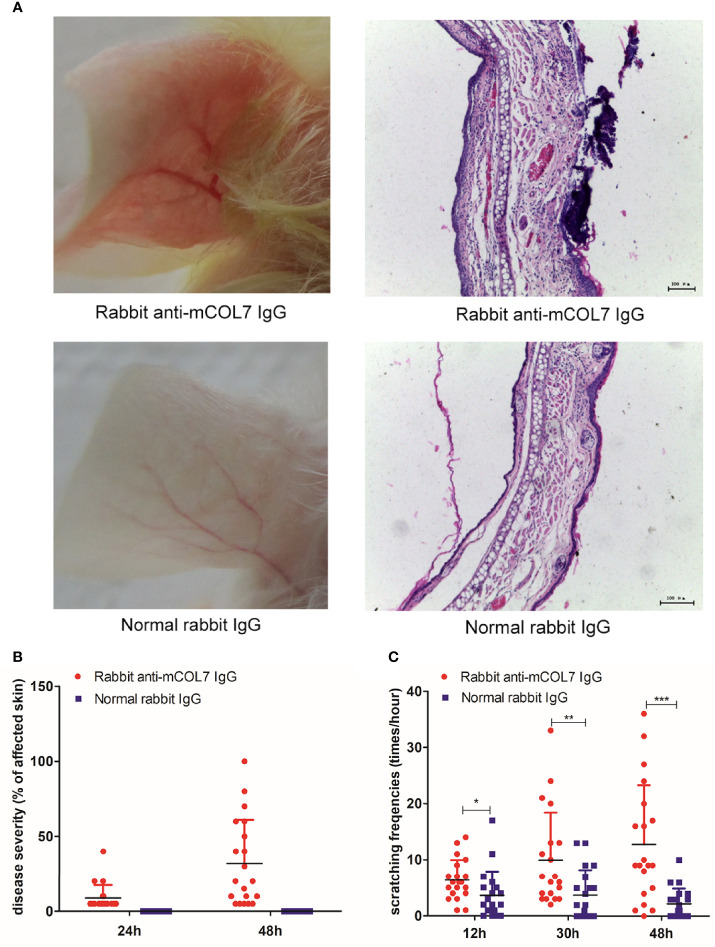
Injection of anti-mCOL7 IgG promotes the frequency of scratching. Female Balb/c mice were injected with 0.5 mg rabbit anti-mCOL7 IgG or control rabbit IgG i.d. into ear skin, and disease severity as well as frequency of scratching was evaluated. **(A)** Representative pictures of ear skin lesions and histology of ear skin of mice treated with rabbit anti-mCOL7 IgG or control rabbit IgG. Histological analysis was performed 48 hours after first antibody injection using H&E staining. **(B)** Mice were scored at 24 and 48 h after the antibody injection, the severity of the disease was measured as the percentage of affected ear skin. **(C)** Scratching behavior was quantified at 12, 30 and 48 h after the antibody injection, and the scratching frequency was presented as times of scratching per hours. Data are derived from 2 independent experiments each performed with 5 mice (10 ears) per experimental group. Bars, 100 μm. Significant differences as determined by unpaired student t-test between anti-mCOL7 IgG-treated ears and control IgG-treated ears are indicated (*p < 0.05, **p < 0.01, ***p < 0.001).

We first investigated whether injection of pathogenic antibodies against mCOL7 could affect the scratching behavior of mice. Injection of rabbit anti-mCOL7 IgG i.d. into the ear induced skin lesions which were first observed at 24 h and further exacerbated by 48 h after the antibody injection (**Figures 1A, B**). By contrast, injection of normal rabbit IgG did not induce any disease.

To quantify scratching behavior, we next determined the frequencies of scratching at 12, 30, and 48 h after injection of antibodies. As compared with mice injected with normal rabbit IgG, mice injected with rabbit anti-mCOL7 IgG showed significantly higher scratching frequencies at all three time points after antibody injection, and the difference increased over time (**Figure 1C**), suggesting that injection of pathogenic antibodies against mCOL7 promoted scratching behavior in anti-mCOL7 IgG-injected mice.

### Topical Application of Dyclonine Prior to Injection of Pathogenic Rabbit IgG Reduces Scratching and Size of Skin Lesions in Experimental EBA

Based on the above findings, we hypothesized that scratching behavior contributes to the development of anti-mCOL7 IgG-mediated tissue damage. To verify the hypothesis, we applied dyclonine hydrochloride, a local anesthetic, topically onto the ear skin before the injection of anti-mCOL7 IgG, and such topical application was maintained until the end of the experiment by repeating the application every 5 hours.

**Figure 2:**
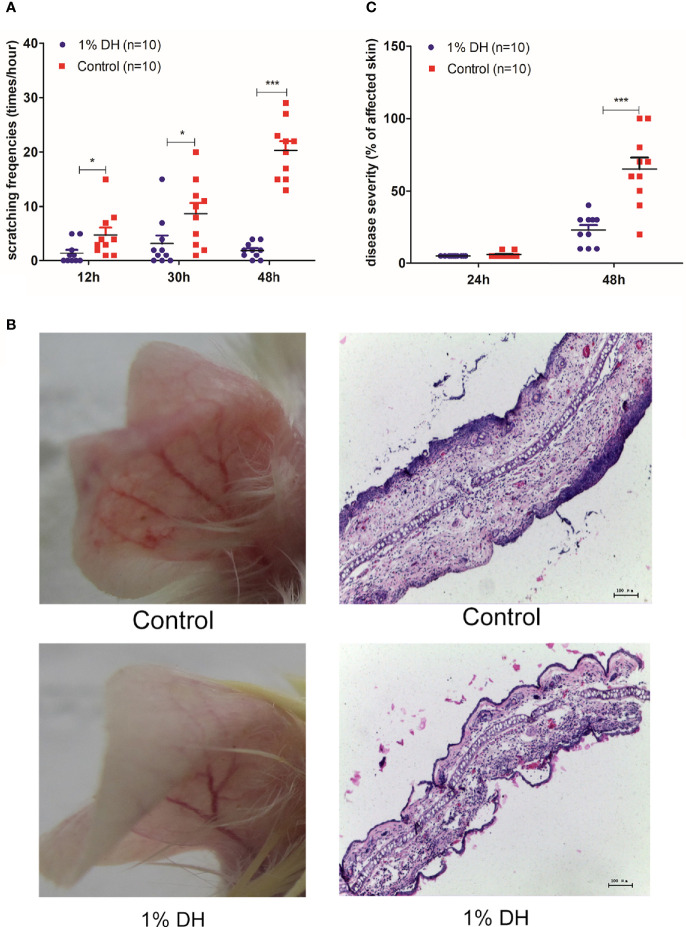
Preventive topical application of dyclonine inhibited scratching and impaired clinical disease manifestation in antibody transfer-induced EBA. Immediately before the injection of anti-mCOL7 IgG, 1% dyclonine or solvent was applied topically onto mouse ears, and such application was maintained every six hours until the end of the experiment. **(A)** Effect of dyclonine hydrochloride on scratching behaviour at 12, 30 and 48 h after the injection of anti-mCOL7 IgG. **(B)** Representative pictures of ear skin lesions and histology in mice injected with rabbit anti-mCOL7 IgG and treated with dyclonine hydrochloride or control solvent. **(C)** Effect of dyclonine hydrochloride on disease severity at 24 and 48 h after the injection of antibodies. This figure shows representative results of one of three independent experiments each performed with 5 mice (10 ears) per experimental group. Bars = 100 μm. Significant differences as determined by unpaired student t test between anti-mCOL7 IgG-treated ears and control IgG-treated ears are indicated (*p < 0.05; ***p < 0.001).

After treatment with dyclonine and subsequent injection of anti-mCOL7 IgG, the scratching frequencies of mouse ears at 12 h, 30 h and 48 h after the injection of anti-mCOL7 IgG antibodies were significantly lower than corresponding values of mouse ears treated with control solvent (**Figure 2A**). This result demonstrates that application of dyclonine prevented mice from anti-mCOL7 IgG-mediated scratching. Furthermore, 48 h after the injection of anti-mCOL7 IgG, dyclonine-treated mice developed significantly milder disease than solvent-treated control mice (**Figures 2B, C**).

### Topical Application of Dyclonine After Injection of Pathogenic Rabbit IgG Has Only a Minor Effect on the Development of Experimental EBA

We next investigated whether application of dyclonine could therapeutically modulate clinical disease manifestation in antibody transfer-induced EBA. At 24 h after the antibody injection, skin lesion was observed in all mice. We then divided these mice into two groups with equal mean values of disease scores. One group was treated with dyclonine, and the other group was treated with solvent. The topical application of dyclonine hydrochloride and control solvent was maintained until the end of the experiment by repeating the application every 5 hours.

**Figure 3:**
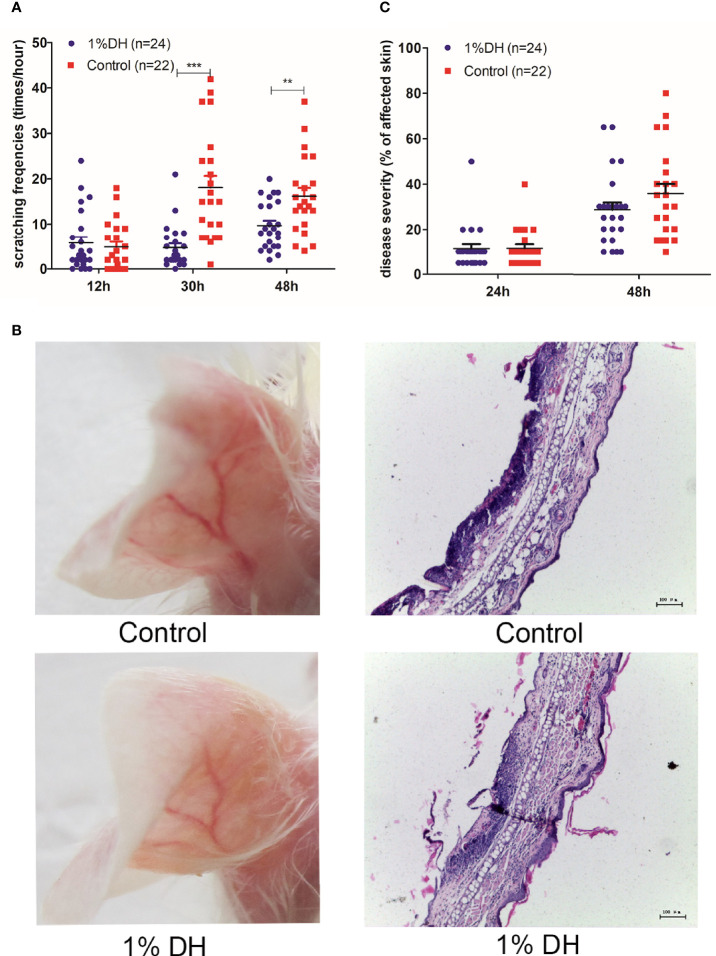
Therapeutic topical application of dyclonine improved itch, but had no impact on clinical disease manifestation in antibody transfer-induced EBA. Twenty-four hours after the injection of anti-mCOL7 IgG, mice were divided into two groups with equal mean value of disease severity. The 1% dyclonine or solvent were applied topically onto mouse ears, respectively, and such application was maintained every six hours until the end of experiment. **(A)** Effect of dyclonine on scratching behaviour at 12, 30 and 48 h after the injection of anti-mCOL7 IgG. **(B)** Representative pictures of skin lesions and histology of ear skin in mice injected with rabbit anti-mCOL7 IgG and treated with dyclonine hydrochloride or control solvent. **(C)** Effect of dyclonine hydrochloride on disease severity at 24 and 48 h after the injection of antibodies. This figure shows results derived from 2 independent experiments.In total, 12 mice (24 ears) treated with 1% dyclonine and 11mice (22 ears) treated with control solvents were used for analysis. Bars, 100 μm. Significant differences as determined by unpaired student t test between anti-mCOL7 IgG-treated ears and control IgG-treated ears are indicated (**p < 0.01, ***p < 0.001).

As expected, the two groups showed no difference in scratching behaviors at 12 h after the injection of anti-mCOL7 IgG because no dyclonine had yet been applied (**Figure 3A**). However, mice treated with dyclonine showed significantly lower scratching frequencies at both 30 h and 48 h after the antibody injection than mice treated with solvent control (**Figure 3A**), demonstrating that the topical application of dyclonine hydrochloride inhibited anti-mCOL7 IgG-mediated scratching.

With regard to the extent of skin lesions, mouse ears treated with dyclonine showed a slight but not significant decreased disease score as compared to mouse ears treated with the solvent control (**Figures 3B, C**), suggesting that topical application of dyclonine had little effect on the extent of the skin disease, if applied 24 h or later after injection of pathogenic IgG.

### Increased Density of Skin Eosinophils and Basophils 48 Hours After Injection of Anti-COL7 IgG

**Figure 4:**
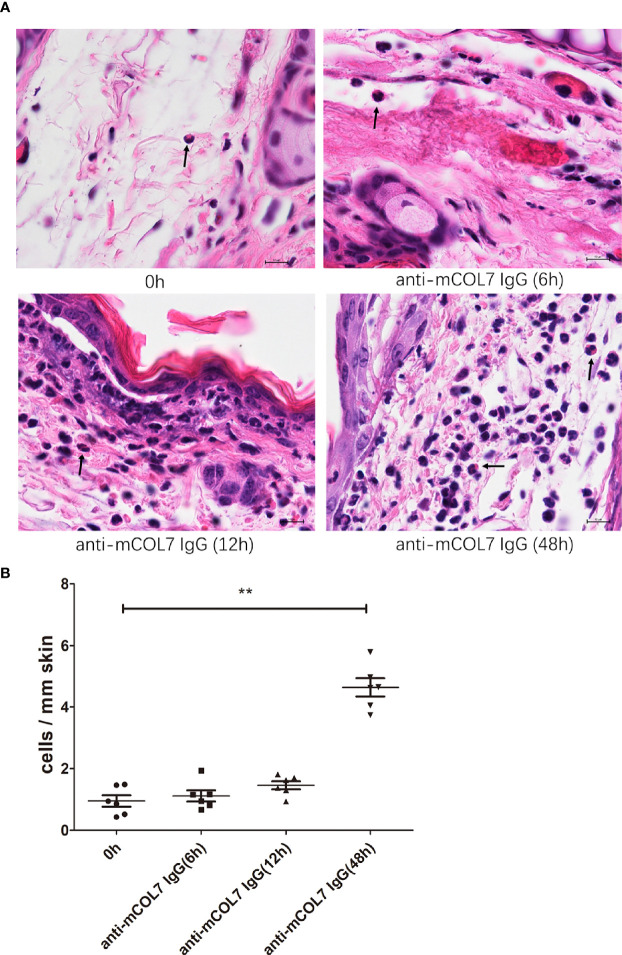
Increased number of skin eosinophils 48 h after injection of anti-COL7 IgG. Female Balb/c mice were injected with 0.5 mg rabbit anti-mCOL7 IgG i.d. into ear skin. Mice were sacrificed and ear skin samples were collected before antibody injection (0 hours) as well as 6, 12 and 48 h after antibody injection. **(A)** Representative micrographs of H&E-stained ear skin sections collected at indicated time points. Black arrows indicate eosinophils. Scale bars = 10 μm. Quantified results of number of eosinophils per mm ear skin section are shown in **(B)** Three mice (6 ears) at each time point were sacrificed and used for analysis. Significant differences as determined by unpaired student t test between anti-mCOL7 IgG-treated ears and control IgG-treated ears are indicated are indicated (**p < 0.01).

Finally, we attempted to identify cells which are responsible for the release of mediators of anti-COL7 IgG-associated itching. Although mast cells (MCs) are the main cellular source of histamine (31) which is the most common mediator of itching (32), our previous results had shown that MCs were dispensable for antibody transfer-induced EBA in mice (26), suggesting that MCs are unlikely the cellular source of mediators of anti-COL7 IgG-associated itching. Given that basophils and eosinophils are also capable to release mediators of itching (31, 32), we next determined the presence of these cells in the ear skin during the development of antibody transfer-induced experimental EBA. As shown in **Figure 4**, eosinophils could be detected in murine skin before the injection of antibodies, and no significant changes in the density of eosinophils was observed in ear skin samples collected at 6 hours and 12 hours after anti-COL7 IgG injection. However, the density of eosinophils was significantly increased at 48 hours after the antibody injection compared to before injection (**Figure 4**). In contrast to eosinophils, basophils could not be detected in healthy murine skin, and skin infiltration of basophils induced by injection of anti-COL7 IgG was rarely observed at 6 or 12 hours after antibody injection. Notably, obvious infiltration of basophils into affected skin was observed at 48 hours after the injection of the anti-COL7 IgG (**Supplementary Figure 2**).

## Discussion

While it has been noticed that itching and scratching are associated with AIBD in patients and mice, it is not clear whether and how scratching contributes to the development of AIBD. In this study, we investigated scratching behavior in an antibody transfer-induced mouse model of EBA. We demonstrate that injection of anti-COL7 IgG promotes scratching in mice. Furthermore, this study shows that topical application of a local anesthetic onto mouse ear before the injection of anti-COL7 IgG inhibits scratching and decreases disease severity. This finding demonstrates that scratching contributes to the development of skin lesions. Interestingly, when a local anesthetic was applied 24 h after injection of antibodies when skin lesions had already appeared, it had little effect on further progression of skin lesions although it did inhibit the scratching. This suggests that scratching is predominantly involved in the initiation phase of antibody transfer-induced experimental EBA but affects only moderately later stages of the disease.

According to the Koebner phenomenon, the appearance of skin lesions as a consequence of trauma has been observed in many skin diseases including psoriasis, atopic dermatitis and vitiligo (33). Since scratch injury is a type of trauma leading to the Koebner phenomenon, the finding of the current study suggests that this concept might also be applied to autoimmune bullous diseases. Regarding the underlying mechanism, it has been suggested that scratch injury leads to Koebner phenomenon in. psoriasis by accelerating the release of chemokines which contribute to the recruitment of IL-17A-producing immune cells and neutrophils (34, 35). Given the essential role of neutrophils in the pathogenesis of experimental EBA (15, 18), it is conceivable that itching and scratching contribute to the development of antibody-transfer induced experimental EBA by recruiting neutrophil at the earlier phase of the disease. Once recruited into the skin and activated by immune complexes, neutrophils contribute to the subsequent tissue damage in two ways. Activated neutrophils adhere to the target tissue and release ROS and proteases which are essential molecules executing tissue damage (15, 18, 19). Additionally, activated neutrophils generate chemotatic factors such as leukotriene B4 and CXCL2 and to recruit more neutrophils into the site of inflammation (20, 36), thus forming a positive feedback loop. Therefore, it is conceivable that scratching contributes to the recruitment of the first wave of neutrophils into the skin. Once the first wave of neutrophils is recruited and activated, scratching is probably dispensable for further disease progression due to the positive feedback loop of neutrophils.

Of specific interest may be the mechanism which leads to the anti-mCOL7 IgG-promoted scratching. Different biological mediators of itch are known of which histamine is the most common one (32). Under both physiological and pathological conditions, mast cells (MCs) are the most relevant cellular sources of histamine (31). Although a role of MCs has been initially suggested in an antibody transfer-induced experimental model of BP (37), more recently it has been demonstrated that MCs are dispensable for antibody transfer-induced EBA in mice (26, 38, 39). Therefore, it is unlikely that MC-derived histamine is the mediator of itch in experimental EBA. It is conceivable that other biological mediators than histamine may contribute to the disease-relevant itch in experimental EBA. One example could be IL-31 which has been suggested to play a role in the development of BP-associated itch (24). The major source of IL-31 in BP are eosinophils (40, 41). Recently, also basophils have been shown to release IL-31 upon activation (42). The current study showed that basophils are not present in the skin under physiological conditions and obvious infiltration of basophils into skin is observed only in the late phase of the anti-mCOL7 IgG-mediated tissue damage. This finding suggests that basophils are not the cellular source of mediators which trigger itching and scratching in the initial phase of experimental EBA. In contrast to basophils, eosinophils are present in the skin under physiological conditions, raising the possibility that eosinophils as the cellular source of the mediator of itching. Thus, further investigation of the role of eosinophils in this model will help to clarify this issue.

One limitation of the current study is that we applied a local transfer model where pathogenic antibodies are applied to a limited site of the ear skin *via* i.d. injection. This allows a precise prediction of the emerging tissue damage and a targeted application of the drug. However, as an acute model, our method is unable to provide any information on the chronic phase of the disease. Other mouse models of EBA generated by either immunization with mCOL7 (5) or systemic and repeatitive injection of anti-mCOL7 IgG (6) could be used for this purpose. Since it is challenging to predict the location of skin lesions in these models, anesthetics could be topically applied to skin regions accessible to scratching, e.g. face, abdomen, tail and feet. Given that only preventive application of dyclonine can inhibit the anti-mCOL7 IgG-mediated skin lesions, treatment of the abovementioned chronic mouse models of EBA with local anesthetics should be applied prior to the binding of pathogenic antibodies onto the antigen.

The finding of the current study might have clinical implications. Topical anesthetics are widely used to relieve pain and itching in numerous medical conditions (43). With regards autoimmune skin blistering diseases, application of anesthetics (e.g, lidocaine) to lesions has been used to reduce pain for patients with mucuous membrane pemphiogoid (44). However, to our knowledge, topical anesthetics have not been utilized to reduce itching and scratching. Our results suggest that topical anesthesia might be a potential treatment to prevent the development of skin lesion by inhibiting itching and scratching.

In conclusion, the current study provides evidence that scratching contributes to the initiation phase of the development of skin lesions in an acute mouse model of EBA. This finding may be of clinical importance because treatment of itch and scratching may decrease the subsequent development of skin lesions.

## Data Availability Statement

The original contributions presented in the study are included in the article/**Supplementary Material**. Further inquiries can be directed to the corresponding author.

## Ethics Statement

The animal study was reviewed and approved by Institutional Animal Care and Use Committee of Xiamen University.

## Author Contributions

XY conceived and supervised the study. LW, XD, GS, QL, and BZ performed experiments and analyzed data. DZ, RL, FP, and XY wrote the manuscript. All authors contributed to the article and approved the submitted version.

## Funding

This work was supported by the National Natural Science Foundation of China (No.81371325) and by the Deutsche Forschungsgemeinschaft: Research Training Group “Modulation of Autoimmunity” (GRK1727), Cluster of Excellence “Inflammation at Interfaces” (EXC 306), Clinical Research Unit “Pemphigoid Diseases” (KFO 303) and the German Center for Lung Research (DZL).

## Conflict of Interest

During the last 3 years, RL has received research funding from MiltenyiBiotec, Biogen, Biotest, Almirall, True North Therapeutics, UCB Pharma, ArgenX, TxCell, Topadur, Incyte and Admirx and fees for consulting or speaking from ArgenX, Immunogenetics, Novartis and Lilly. DZ has received support through research and development grants as well as for consulting or lectureing from Biotest, Fresenius, MiltenyiBiotec, Roche Pharma Inc., Biogen, Abbvie Inc., UCB Pharma, Janssen, Euroimmun Inc., Dompe, Novartis and ArgenX.

The remaining authors declare that the research was conducted in the absence of any commercial or financial relationships that could be construed as a potential conflict of interest.

## Publisher’s Note

All claims expressed in this article are solely those of the authors and do not necessarily represent those of their affiliated organizations, or those of the publisher, the editors and the reviewers. Any product that may be evaluated in this article, or claim that may be made by its manufacturer, is not guaranteed or endorsed by the publisher.
