# Supplementary material for: Preventive but Not Therapeutic Topical Application of Local Anesthetics Can Inhibit Experimental Epidermolysis Bullosa Acquisita in Mice
Source: Front Immunol. 2021 Oct 12;12:750160. doi: 10.3389/fimmu.2021.750160 (PMC8546209; doi:10.3389/fimmu.2021.750160)
Supplement: Supplementary file 1 [file DataSheet_1.pdf]

Supplementary information

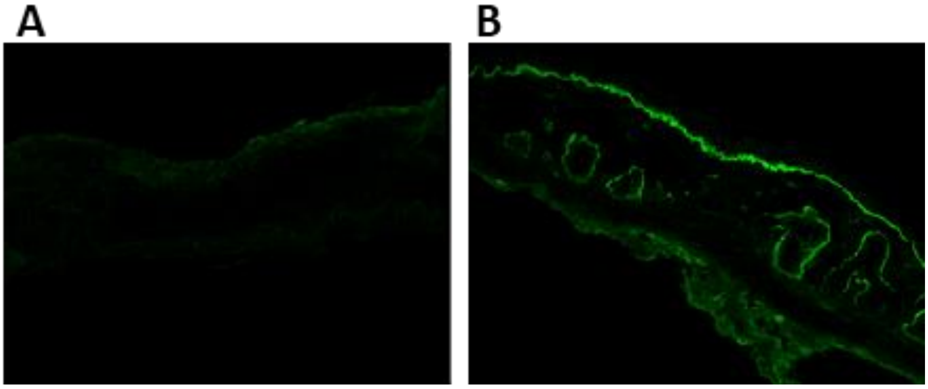

Supplementary figure 1: Specific binding of rabbit anti-COL7 IgG to the dermal-epidermal junction zone of murine skin. Cryosections of murine skin were incubated with 0.1 mg/ml control rabbit IgG (A) or rabbit anti-mCOL7 IgG (B). Subsequently, the binding of rabbit IgG was visualized by staining with Alex488-goat anti-rabbit IgG.

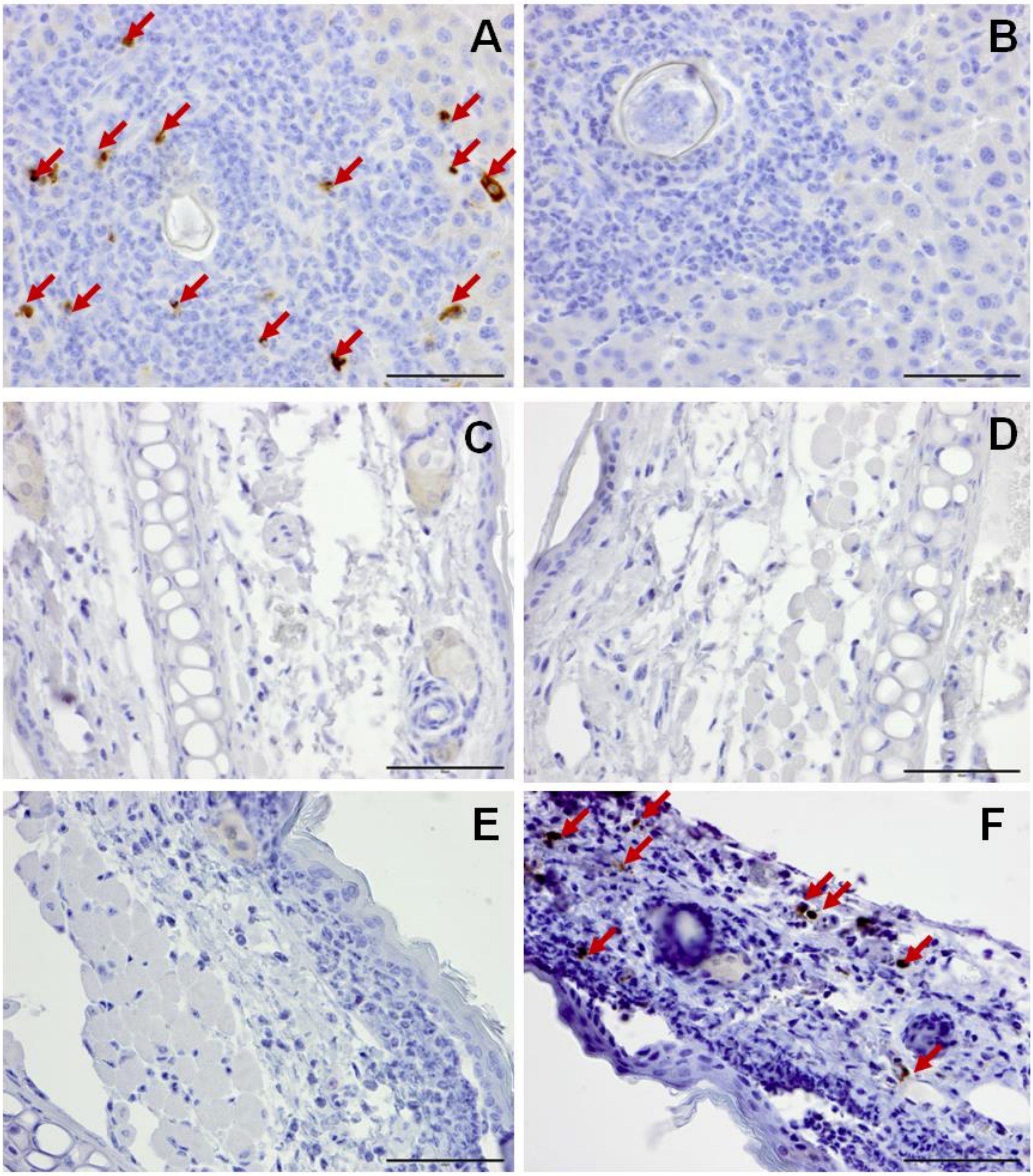

Supplementary figure 2. The presence of basophils in the development of anti-COL7 IgG transfer-induced experimental EBA. Female Balb/c mice were injected with 0.5 mg rabbit anti-mCOL7 IgG i.d. into ear skin. Mice were sacrificed and ear skin samples were collected before antibody injection (0 hours) as well as 6, 12 and 48 h after antibody injection. Liver tissue sections from mice infected with the parasitic worm *Schistosoma mansoni* were used as positive controls for immunochemistry staining with (A) the anti-mMCP8 IgG or (B) isotype control IgG. Representative micrographs of anti-mMCP8 IgG-stained ear skin sections collected at (C) 0 hour, (D) 6 hours, (E) 12 hours and (F) and 48 hours after the anti-mCOL7 IgG injection. Red arrows indicate anti-MCP8-positive basophils. Scale bars = 50 μm.
